# Relative Validity of a 24-h Recall in Assessing Intake of Key Nutrients in a Cohort of Australian Toddlers

**DOI:** 10.3390/nu10010080

**Published:** 2018-01-12

**Authors:** Elizabeth Beaton, Janine Wright, Gemma Devenish, Loc Do, Jane Scott

**Affiliations:** 1 School of Public Health, Curtin University, Perth, WA 6102, Australia; elizabeth.beaton@postgrad.curtin.edu.au (E.B.); j.wright@exchange.curtin.edu.au (J.W.); gemma.devenish@curtin.edu.au (G.D.); 2 Australian Research Centre for Population Oral Health, University of Adelaide, Adelaide, SA 5000, Australia; loc.do@adelaide.edu.au

**Keywords:** 24-h dietary recall, food record, nutrient intake, relative validity, toddlers

## Abstract

There is limited information concerning the dietary intake of toddlers in Australia. Consequently, there is a need for studies investigating toddler intake that use dietary assessment measures that are valid and place a low participant burden on caregivers. The aim of this study was to determine the relative validity of a single 24-h dietary recall (24HR) in measuring the intake of five nutrients in a cohort of Australian toddlers compared to a combined 24HR and 2-day estimated food record (2DFR). A single 24HR and a 2DFR were collected from a cohort of Australian toddlers (*n* = 699) at approximately 12 months of age as part of the Study of Mothers’ and Infants’ Life Events affecting oral health (SMILE) project. Relative validity of one day of dietary data (24HR) in assessing intake of energy, protein, calcium, iron, and added sugars was tested against three days of dietary data (24HR + 2DFR) using paired *t*-tests, Bland–Altman analysis, cross-classification, and weighted Kappa statistic. Classification analysis found good agreement between the 24HR and 24HR + 2DFR for all nutrients with the percentage classified in the same tertile at 57.9% and above. The weighted Kappa statistic found acceptable agreement for all nutrients. This study suggests that a 24HR is a valid assessment tool for estimating the relative intake of energy, protein, calcium, iron, and added sugars among Australian toddlers at the individual level.

## 1. Introduction

In Australia, national dietary data collection has concentrated on infant feeding and intake of children aged 2 years and older, and there is limited dietary information available that examines the intake of toddlers (12–24 months) [1,2]. The toddler period is important as it sees the transition from a predominantly single-food diet (i.e., breast milk or formula) to a more diverse dietary pattern described as family foods [3]. This age group is associated with increased nutrient demands as children grow and develop, but existing Australian research has found high intake of discretionary foods amongst toddlers, often in conjunction with poor vegetable consumption [4,5,6].

Specific challenges associated with investigating toddler intake include high levels of plate waste, frequent consumption of small amounts of food, and the need for proxy reporting completed by a parent or caregiver [7,8,9]. To minimise misreporting and maximise participation, the dietary assessment method chosen must take both these challenges and participant burden on the part of the caregiver into account. In addition, in the absence of the ability to determine absolute validity, the relative validity of the assessment method to measure actual intake must be established [10].

The food frequency questionnaire (FFQ), 24-h dietary recall (24HR), and multiple-day food record (FR) have been used to assess toddler intake at a population level overseas [7,11,12] and in smaller studies in Australia [9,13,14,15]. The FFQ has been used to assess the intake of pre-school-aged children (12 months to 5 years), primarily due to low participant burden, cost effective application, and its appropriateness for assessing usual dietary intake in large populations [16,17]. However, relative validity of the FFQ in assessing the intake of pre-school-aged children varies with overestimation of energy, nutrients, and food groups found in several validation studies when compared to the reference method [8,18,19]. The FR is associated with greater accuracy, and this is demonstrated by its frequent use as the reference method in validation studies [8,19,20,21,22,23]. However, FR carries a high participant burden and its value as a measure of usual diet is limited by the potential for the act of recording to alter the types and amounts of food consumed [16,17].

The 24HR relies on trained interviewers and portion size estimation skills for accuracy [12,16,17]. In addition, it is not considered to provide a reliable estimate of day-to-day variation [24]. However, within-subject variation has been found to be lower than between-subject variation in children aged 12 months [25]. Lanigan et al. [26] suggest this may be due to a general lack of variety in toddler diets which can be comprised of fewer foods than adults and older children. Consequently, this limitation may be of less concern when estimating usual diet in children aged 12 months than among older age groups.

To date, the validity of the 24HR in assessing the intake of young children has been the subject of only one study [21]. Validity was found to be poor with energy and nutrient intake significantly overestimated in comparison to a 3-day weighed FR, but the conclusions of this study are limited because a single statistical test of relative validity was used [21]. As a quantitative retrospective measure of actual intake with a relatively low participant burden, the 24HR is a dietary assessment tool that warrants further investigation within the Australian toddler population.

The aim of this study was to determine the relative validity of a single 24HR in estimating the intake of key nutrients (energy, protein, calcium, iron, and added sugars) in a cohort of Australian toddlers, compared to a combined 24HR and 2-day FR (24HR + 2DFR).

## 2. Materials and Methods

This is a cross-sectional analysis of dietary data collected as part of the Study of Mothers’ and Infants’ Life Events Affecting Health (SMILE), a longitudinal birth cohort study. A cohort of socioeconomically diverse newborns in South Australia were recruited and followed from birth to 24–30 months. Between July 2013 and August 2014, a total of 2147 mothers and 2181 infants (including 34 pairs of twins) were recruited from three maternity hospitals in Adelaide, Australia. Recruitment occurred on postnatal wards within 48-h of giving birth. Invitations to participate were extended to all new mothers whose English competency allowed them to sufficiently understand the study description and instruction. Exclusions were those mothers who intended to move out of the Adelaide area within a year.

Mothers received a written and verbal description of the study and were advised that their participation was voluntary and that they could decline to participate and withdraw from the study at any time without prejudice. Participants provided signed informed consent. Ethics approval for the SMILE study was granted by the Southern Adelaide Clinical Human Research Ethics Committee (HREC/50.13: approved 28 February 2013) and the South Australian Women and Children Health Network (HREC/13/WCHN/69: approved 7 August 2013). Reciprocal ethics approval was also granted by the Curtin University Human Research Ethics Committee (HREC/155/2013: approved 10 October 2013).

Baseline socio-demographic information was collected via a questionnaire completed by mothers at the time of recruitment. Dietary data were collected from mothers when children reached 12 months of age using a 24HR and a 2DFR. The three days of dietary collection were non-consecutive, within a 10-day period and contained two weekdays and one weekend day. Prior to dietary data collection, participants were sent a food diary booklet containing instructions for completion, a 1-day detailed example, photos of food portion sizes and examples of household measures to assist mothers in estimating amounts of food consumed for use with both data collection methods. An accompanying cover letter advised mothers that a researcher would be contacting them to conduct the 24HR and to explain how to complete the 2DFR. The 24HR of their child’s intake was completed via telephone with a trained dietitian using the five-step multipass method [27]. Following this interview, mothers were allocated two days to record their child’s intake using the food diary booklet and requested to return the completed 2DFR in a reply-paid envelope.

Paired dietary data (24HR and 2DFR) were entered together as a 3-day food record into FoodWorks 8 (Xyris Software, 2012–2016, Brisbane, QSL, Australia) and analysed using the AUSNUT 2011–2013 food composition database [28]. Nutrient information for food items recorded but not listed in the AUSNUT database were added using information from product nutrition information panels and manufacturer websites. Dietary data were double-entered by a team of nutritionists who had received standardised training, using a detailed data entry protocol.

A second food record was created for each study participant in FoodWorks 8 by removing the 2DFR, leaving data from the 24HR only. This resulted in a single day (24HR) and three days (24HR + 2DFR) of dietary information available for each participant. Food records were imported into SPSS version 23.0 (SPSS Inc., Chicago, IL, USA) via Microsoft Access for statistical analysis. To account for misreporting, a plausible energy intake was determined for each child by calculating age (using child’s date of birth and the 24HR completion date) and reference values for gender-specific estimated energy requirement (EER) [29]. Reference values were used, as current weights of the children were unavailable. Children were deemed to have an implausible energy intake if they had an average daily energy intake below 0.54 or above 1.46 for their reference EER [30], and subsequently were excluded from further analysis.

The nutrients explored in this study included energy, protein, calcium, iron, and added sugars. Added sugars is defined according to AUSNUT 2011–2013 as including added forms of dextrose, fructose, sucrose, lactose, sugar syrups, and fruit syrups, but not the sugar components of honey and fruit juice [28].

Descriptive statistics were run to identify outliers, improbable intakes and test for normality. The level of significance was set at *p* < 0.05. Raw data were normally distributed with the exceptions of iron and added sugars. The 24HR and 24HR + 2DFR iron and added sugars variables were log-transformed to produce a normal distribution and these transformed variables were used for further validity analysis.

Multiple tests were conducted to compare the intake of these nutrients between the single day (24HR) and three days (24HR + 2DFR) of dietary data for those children with plausible intakes. Paired *t*-tests were conducted to test for differences in mean intake between one and three days of dietary data at the group level. Bland–Altman analysis was performed to investigate the difference in nutrient intake between one day and three days against the mean of the two methods [31]. Graphs were assessed visually and strength of agreement at individual level interpreted as good, fair, or poor based on the difference between the limits of agreement (LOA) [32]. At the group level, agreement was assessed by the mean bias. Linear regression analysis was performed for each nutrient to test if the slope of mean bias was significantly different to 0, thus testing for the presence of proportional bias [33].

At the individual level, cross-classification analysis was conducted to test for agreement between the two methods in ranking participants correctly [10]. Participants were classified into tertiles based on their intake for each of the nutrients. The percentage of participants correctly classified into the same tertile by each dietary data set was calculated along with the percentage grossly misclassified. Gross misclassification was defined as the 24HR categorising the nutrient intake in the highest tertile when the 24HR + 2DFR categorised it in the lowest tertile, or vice versa. The weighted Kappa statistic was also calculated to determine the level of agreement beyond chance between the 24HR and 24HR + 2DFR. Interpretation criteria describe acceptable agreement as values between 0.2 and 0.6, and values of 0.61 and greater to be considered good agreement [10].

## 3. Results

Of the 1919 mothers who were mailed a food record when their child reach 12 months of age, 1165 completed a 24HR and 844 returned the 2DFR. Three days of complete and usable dietary data were available for 828 children. Following exclusion of those children identified as having implausible intakes (*n* = 129), the study population was reduced to 699 children with a mean age of 12.85 months. See Supplementary Table S1 for the demographic characteristics of the mother–child dyads.

Mean ± standard deviation (SD) and median intakes of each nutrient for both methods, skewness, and mean differences in dietary intakes between 1-day and 3-days are presented in Table 1. As is often found in dietary data, raw values showed slight positive skew with the exception of a single variable (24HR + 2DFR energy) [34]. The 24HR provided higher estimated intakes of all nutrients, and a small but statistically significant difference in intake between the 24HR and the 24HR + 2DFR was found for energy (66 kJ, *p* = 0.008) and added sugars (0.18 g, *p* < 0.001).

**Figure 1:**
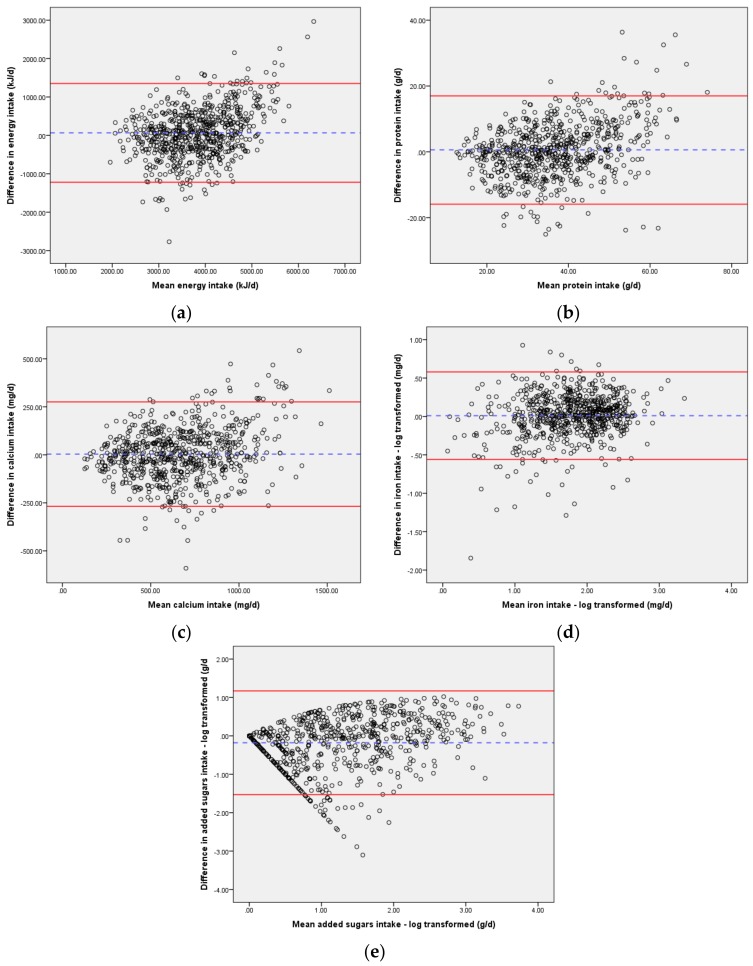
Bland–Altman plots of agreement between dietary intakes reported in the 24HR and 24HR + 2DFR in Australian toddlers aged 12 months (*n* = 699): (**a**) Energy intake; (**b**) Protein intake; (**c**) Calcium intake; (**d**) Iron intake; (**e**) Added sugars intake. Difference in intake for each plot (*y*-axis) is 24HR − (24HR + 2DFR). The plots show the mean difference (---) and the limits of agreement (―) for each nutrient. kJ/d: kJ/day; g/d: g/day; mg/d: mg/day.

Table 2 presents the results from the Bland–Altman analysis and shows mean difference and LOA for each nutrient. Limits of agreement were wide for energy, protein, and iron, showing poor agreement between the two methods (Figure 1). For calcium and added sugars, the LOA were narrower, showing fair to poor agreement between the two methods at an individual level. Linear regression analysis demonstrated the slope of the mean bias for each nutrient was significantly different to 0, identifying proportional bias (indicating differences between the 24HR and 24HR + 2DFR increased as the mean intake increased).

When ranking participants according to their intake using weighted Kappa statistic, acceptable agreement beyond chance was found between the 24HR and the 24HR + 2DFR for energy, protein, calcium, and added sugars, with good agreement for iron [10] (Table 3).

Cross-classification analysis also found good agreement between the 24HR and 24HR + 2DFR with the percentage classified in the same tertile at 60.8% and above for energy, protein, calcium, and iron, and 57.9% for added sugars. Gross misclassification was 3.6% or less for all nutrients, which was well within the acceptable level of less than 10% [10] (Table 3).

## 4. Discussion

Relative validity of the 24HR in assessing the intake of five nutrients by Australian toddlers was tested at group and individual level using multiple tests of validity. At the group level, the 24HR was found to significantly overestimate group mean intake of energy and added sugar when compared to the 24HR + 2DFR. However, these differences are of little clinical significance given the small absolute values these differences represented. The study by Fisher et al. [21] also found a single 24HR to significantly overestimate intake of energy and nutrients in pre-school-aged children when tested against a 3-day FR but actual differences in estimated intake were much greater. Fisher et al. [21] found the 24HR to overestimate energy intake in toddlers (*n* = 77) by 29%. In contrast, this study determined energy intake to be overestimated by 1.7% when tested against three days of dietary data.

Bland–Altman analysis has been described as a “more rigorous approach to assessing agreement as it accounts for correlated error” [33] (p. 263) and did not support results obtained by the other tests of relative validity. Nevertheless, the wide limits of agreement (LOA) suggesting poor agreement between the 24HR and the 24HR + 2DFR are similar to those found in other validation studies in children [8,19] and adults [35]. Watson et al. [32] assessed the validity of a FFQ in Australian children and adolescents and found wide LOA for nutrients and strong trends of greater differences with increasing intakes. Similarly, the presence of proportional bias for each nutrient in this study showed that the difference was greater at higher levels of intake, suggesting that the 24HR is less suitable for estimating intakes at the group level.

The cross-classification analysis for nutrients produced better results than the two validation studies in young children that reported cross-classification [8,20]. Gross misclassification was well within acceptable levels [10], being highest for added sugars at 3.6% and below 2.7% for energy, calcium, protein, and iron, suggesting that one day of dietary data (24HR) can successfully rank children according to their intake of energy, protein, calcium, iron, and added sugars when compared to three days of dietary data (24HR + 2DFR). This is supported by the weighted Kappa statistic that found acceptable agreement beyond chance for all nutrients when ranking children using the 24HR and 24HR + 2DFR.

The dietary intake data analysed in this study were collected in the SMILE study, which is a birth cohort study designed to examine a wide range of determinants influencing oral health in early childhood [36]. From a dietary perspective, the primary dietary explanatory variable of interest in the SMILE study is intake of added sugars. However, added sugars have not been investigated in previous validation studies involving pre-school-aged children, making comparison with this analysis impossible [8,19,20,21,23,37].

Because of high day-to-day variation in intake, numerous days of intake data are typically required to estimate usual dietary intake. The number of days varies according to the individual nutrient and the desired precision of estimate [38,39]. According to Nelson et al., the number of days is “lowest for nutrients that appear regularly in the diets of some subjects but not others (such as sugars) and highest for nutrients that appear in large amounts only occasionally in almost all subjects’ diets (such as carotene)” [38] (p. 164). In addition, the number of days will vary according to an individual’s age with fewer days generally being required for toddlers than for older children and adults [25,39]. Compared with older children, within-subject variation has been found to be lower than between-subject variation in children aged 12 months [25], probably as a result of children of this age consuming diets which are comprised of fewer foods than older children and adults [26]. In support of the findings of this study related to added sugars, Erkola et al. reported that only one to two days of dietary data was required to assess intake of total sugars and sucrose to a high level of precision in children aged 12 months [25].

The results of this analysis suggest that data from the single 24HR will be suitable to assess the relative intake of added sugars of participants in the SMILE study in order to explore the association of ranked intake of added sugars and early childhood caries. This will allow a greater number of subjects to be included in the analysis of the SMILE data, given the larger number of subjects with a 24HR than those with a complete 24HR + 2DFR. The fact that only three-quarters of mothers who completed the 24HR also returned a completed 2DFR is evidence of the added participant burden associated with completing a FR [34].

The results of this study are similar to, if not stronger than other studies that concluded acceptable to good validity in their validation studies estimating nutrient intake in pre-school-aged children [8,19,23]. A strength of this study was the use of four statistical tests of validity compared to other validation studies in pre-school aged children that used fewer tests [8,18,19,20,21,22,23,37]. The large cohort size and the exclusion of children with implausible intakes are additional strengths. Limitations of this study include the absence of external biomarker data to validate results and the return of complete dietary data from less than 50% of participants. As mothers from low socioeconomic groups were intentionally oversampled as part of the SMILE study design, this sample is still considered to be generally representative of the population from which it was drawn [30].

## 5. Conclusions

The 24HR demonstrated good relative validity in estimating the intake of energy, protein, calcium, iron, and added sugars in statistical tests of validity at an individual level when compared to the 24HR + 2DFR. Paired *t*-tests found statistically significant differences for energy and added sugars between the 24HR and the 24HR + 2DFR, but the actual differences were very small. The bias detected using Bland–Altman analysis indicates difference in intake between the two methods increased with higher levels of intake. However, the 24HR performed well at an individual level with good validity demonstrated in cross-classification and weighted K statistical analyses. Findings from this study indicate that a single day of dietary data, collected via 24HR, may be a valid method for estimating relative intake of energy, protein, iron, calcium, and added sugars at an individual level in Australian toddlers.

## Figures and Tables

**Table 1:** Average daily intake of key nutrients reported in the 24HR and the 24HR + 2DFR, and differences in intakes between the 24HR and 24HR + 2DFR in Australian toddlers aged 12 months (*n* = 699).

	24HR			24HR + 2DFR			Difference ^1^	
Nutrient	Mean (±SD)	Median	Skewness	Mean (±SD)	Median	Skewness	Mean (±SD)	*p*^2^
Energy (kJ)	3849 (928)	3793	0.461	3783 (701)	3827	−0.174	65.6 (656.1)	0.008
Protein (g)	36.8 (13.5)	35.5	0.586	36.3 (10.8)	35.6	0.223	0.6 (8.4)	0.078
Calcium (mg)	665.9 (282.1)	627.8	0.528	662.1 (247.2)	651.9	0.214	3.78 (138.8)	0.471
Iron (mg)	6.9 (3.7)	6.5	1.398	6.7 (3.3)	6.0	1.096	0.22 (1.88)	0.002
Iron (mg) ^3^							0.01 (0.29)	0.257
Added sugars (g)	4.5 (6.8)	2.0	3.095	4.5 (5.2)	2.6	2.431	−0.003 (4.77)	0.987
Added sugars (g) ^3^							−0.18 (0.69)	<0.001

^1^ Mean difference of 24HR − (24HR + 2DFR); ^2^ Paired *t*-Test used to compare difference in methods, significant at *p* < 0.05; ^3^ Log-transformed values used for tests of validity; 24HR: 24-h dietary recall; 2DFR: 2-day estimated food record; SD: standard deviation.

**Table 2:** Bland–Altman statistics comparing intake of key nutrients from the 24HR and 24HR + 2DFR in Australian toddlers aged 12 months (*n* = 699).

Nutrient	24HR vs. 24HR + 2DFR	
	Mean Difference ^1^ (95% LOA)	Slope of Bias ^2^
Energy (kJ)	65.6 (−1220.4, 1351.7)	0.326
Protein (g)	0.6 (−15.9, 17.0)	0.259
Calcium (mg)	3.8 (−268.3, 275.8)	0.141
Iron (mg) ^3^	0.01 (−0.56, 0.58)	0.100
Added sugars (g) ^3^	−0.18 (−1.53, 1.17)	0.209

^1^ Mean difference of 24HR − (24HR + 2DFR); ^2^ For all slope of bias, *p* < 0.001; ^3^ data log-transformed. LOA: limits of agreement.

**Table 3:** Cross-classification for agreement between the 24HR and 24HR + 2DFR for average intake of nutrients and weighted Kappa statistic in Australian toddlers aged 12 months (*n* = 699).

	24HR vs. 24HR + 2DFR		
Nutrient	% Correctly Classified ^1^	% Grossly Misclassified ^2^	*κ*
Energy (kJ)	60.8	2.6	0.412
Protein (g)	67.8	2.7	0.517
Calcium (mg)	70.8	0.6	0.562
Iron (mg) ^3^	77.3	1.0	0.659
Added sugars (g) ^3^	57.9	3.6	0.369

^1^ % correctly classified = percentage of children classified into the same tertile by the 24HR and the 24HR + 2DFR. If the two methods were completely unrelated, 33.3% correct classification would be expected; ^2^ % grossly misclassified = percentage of children classified into the highest tertile by the 24HR when the 24HR + 2DFR classified them into the lowest tertile, and vice versa. If the two methods were completely unrelated, 22.2% gross misclassification would be expected; ^3^ Log-transformed values used for tests of validity.

## Supplementary Materials

The following are available online at http://www.mdpi.com/2072-6643/10/1/80/s1, Table S1: Characteristics of mother–child dyads with complete dietary data and of the plausible and implausible energy intake subsets.

## References

[1] Australian Institute of Health and Welfare (2011). 2010 Australian National Infant Feeding Survey: Indicator Results.

[2] Australian Bureau of Statistics (2014). Australian Health Survey: Nutrition First Results—Food and Nutrients, 2011–2012.

[3] Nicklaus S. (2011). Children’s acceptance of new foods at weaning. Role of practices of weaning and of food sensory properties. Appetite.

[4] Byrne R., Magarey A., Daniels L. (2014). Food and beverage intake in Australian children aged 12–16 months participating in the NOURISH and SAIDI studies. Aust. N. Z. J. Public Health.

[5] Chan L., Magarey A.M., Daniels L.A. (2010). Maternal Feeding Practices and Feeding Behaviors of Australian Children Aged 12–36 Months. Matern. Child Health J..

[6] Lioret S., McNaughton S.A., Spence A.C., Crawford D., Campbell K.J. (2013). Tracking of dietary intakes in early childhood: The Melbourne InFANT Program. Eur. J. Clin. Nutr..

[7] Lennox A., Sommerville J., Ong K., Henderson H., Allen R. (2013). Diet and Nutrition Survey of Infants and Young Children, 2011.

[8] Watson E.O., Heath A.-L.M., Taylor R.W., Mills V.C., Barris A.C., Skidmore P.M. (2015). Relative validity and reproducibility of an FFQ to determine nutrient intakes of New Zealand toddlers aged 12–24 months. Public Health Nutr..

[9] Webb K., Rutishauser I., Knezevic N. (2008). Foods, nutrients and portions consumed by a sample of Australian children aged 16–24 months. Nutr. Diet..

[10] Lombard M.J., Steyn N.P., Charlton K.E., Senekal M. (2015). Application and interpretation of multiple statistical tests to evaluate validity of dietary intake assessment methods. Nutr. J..

[11] Emmett P.M., Jones L.R. (2014). Diet and growth in infancy: Relationship to socioeconomic background and to health and development in the Avon Longitudinal Study of Parents and Children. Nutr. Rev..

[12] Ziegler P., Briefel R., Clusen N., Devaney B. (2006). Feeding Infants and Toddlers Study (FITS): Development of the FITS Survey in Comparison to Other Dietary Survey Methods. J. Am. Diet. Assoc..

[13] Amezdroz E., Carpenter L., O’Callaghan E., Johnson S., Waters E. (2015). Transition from milks to the introduction of solid foods across the first 2 years of life: Findings from an Australian birth cohort study. J. Hum. Nutr. Diet..

[14] Campbell K., Hesketh K., Crawford D., Salmon J., Ball K., McCallum Z. (2008). The Infant Feeding Activity and Nutrition Trial (INFANT) an early intervention to prevent childhood obesity: Cluster-randomised controlled trial. BMC Public Health.

[15] Daniels L.A., Magarey A., Battistutta D., Nicholson J.M., Farrell A., Davidson G., Cleghorn G. (2009). The NOURISH randomised control trial: Positive feeding practices and food preferences in early childhood—A primary prevention program for childhood obesity. BMC Public Health.

[16] Rutishauser I.H.E. (2005). Dietary intake measurements. Public Health Nutr..

[17] Thompson F.E., Subar A.F., Coulston A., Boushey C.J., Ferruzzi M.G. (2013). Dietary Assessment Methodology. Nutrition in the Prevention and Treatment of Disease.

[18] Bel-Serrat S., Mouratidou T., Pala V., Huybrechts I., Börnhorst C., Fernández-Alvira J.M., Hadjigeorgiou C., Eiben G., Hebestreit A., Lissner L. (2014). Relative validity of the Children’s Eating Habits Questionnaire-food frequency section among young European children: The IDEFICS Study. Public Health Nutr..

[19] Vereecken C., Covents M., Maes L. (2010). Comparison of a food frequency questionnaire with an online dietary assessment tool for assessing preschool children’s dietary intake. J. Hum. Nutr. Diet..

[20] Cheng G., Hilbig A., Drossard C., Alexy U., Kersting M. (2013). Relative validity of a 3 d estimated food record in German toddlers. Public Health Nutr..

[21] Fisher J.O., Butte N.F., Mendoza P.M., Wilson T.A., Hodges E.A., Reidy K.C., Deming D. (2008). Overestimation of infant and toddler energy intake by 24-h recall compared with weighed food records. Am. J. Clin. Nutr..

[22] Huybrechts I., De Backer G., De Bacquer D., Maes L., De Henauw S. (2009). Relative Validity and Reproducibility of a Food-Frequency Questionnaire for Estimating Food Intakes among Flemish Preschoolers. Int. J. Environ. Res. Public Health.

[23] Marriott L.D., Inskip H.M., Borland S.E., Godfrey K.M., Law C.M., Robinson S.M. (2009). What do babies eat? Evaluation of a food frequency questionnaire to assess the diets of infants aged 12 months. Public Health Nutr..

[24] Nelson M., Bingham S., Margetts B.M., Nelson M. (2009). Assessment of food consumption and nutrient intake. Design Concepts in Nutritional Epidemiology.

[25] Erkkola M., Kyttälä P., Takkinen H.-M., Kronberg-Kippilä C., Nevalainen J., Simell O., Ilonen J., Veijola R., Knip M., Virtanen S.M. (2011). Nutrient intake variability and number of days needed to assess intake in preschool children. Br. J. Nutr..

[26] Lanigan J.A., Wells J.C.K., Lawson M.S., Cole T.J., Lucas A. (2004). Number of days needed to assess energy and nutrient intake in infants and young children between 6 months and 2 years of age. Eur. J. Clin. Nutr..

[27] Australian Bureau of Statistics (2014). Australian Health Survey: Users’ Guide, 2011–2013.

[28] Food Standards Australia New Zealand AUSNUT 2011–2013—Australian Food Composition Database. http://www.foodstandards.gov.au/science/monitoringnutrients/ausnut/foodnutrient/Pages/default.aspx.

[29] National Health and Medical Research Council and Ministry of Health (2006). Nutrient Reference Values for Australia and New Zealand.

[30] Scott J., Davey K., Ahwong E., Devenish G., Ha D., Do L. (2016). A Comparison by Milk Feeding Method of the Nutrient Intake of a Cohort of Australian Toddlers. Nutrients.

[31] Bland M.J., Altman D.G. (1986). Statistical Methods for Assessing Agreement between Two Methods of Clinical Measurement. Lancet.

[32] Watson J.F., Collins C.E., Sibbritt D.W., Dibley M.J., Garg M.L. (2009). Reproducibility and comparative validity of a food frequency questionnaire for Australian children and adolescents. Int. J. Behav. Nutr. Phys. Act..

[33] Magarey A., Golley R., Spurrier N., Goodwin E., Ong F. (2009). Reliability and validity of the Children’s Dietary Questionnaire: A new tool to measure children’s dietary patterns. Int. J. Pediatr. Obes..

[34] Dietary Assessment Primer. https://dietassessmentprimer.cancer.gov/learn/distribution.html.

[35] Barrett J.S., Gibson P.R. (2010). Development and Validation of a Comprehensive Semi-Quantitative Food Frequency Questionnaire that Includes FODMAP Intake and Glycemic Index. J. Am. Diet. Assoc..

[36] Do L., Scott J., Thomson W., Stamm J., Rugg-Gunn A., Levy S., Wong C., Devenish G., Ha D., Spencer A. (2014). Common risk factor approach to address socioeconomic inequality in the oral health of preschool children—A prospective cohort study. BMC Public Health.

[37] Mejía-Rodríguez F., Neufeld L.M., García-Guerra A., Quezada-Sanchez A.D., Orjuela M.A. (2014). Validation of a Food Frequency Questionnaire for Retrospective Estimation of Diet During the First 2 Years of Life. Matern. Child Health J..

[38] Nelson M., Black A.E., Morris J.A., Cole T.J. (1989). Between- and within-subject variation in nutrient intake from infancy to old age: Estimating the number of days required to rank dietary intakes with desired precision. Am. J. Clin. Nutr..

[39] Institute of Medicine (2000). Dietary Reference Intakes: Applications in Dietary Assessment.

