# Supplementary material for: Relative Validity of a 24-h Recall in Assessing Intake of Key Nutrients in a Cohort of Australian Toddlers
Source: Nutrients. 2018 Jan 12;10(1):80. doi: 10.3390/nu10010080 (PMC5793308; doi:10.3390/nu10010080)
Supplement: Supplementary file 1 [file nutrients-10-00080-s001.pdf]

Supplementary Materials

Table S1. Characteristics of mother-child dyads with complete dietary data and of the subsets with plausible and implausible energy intake.

	**Total**		**Plausible**		**Implausible**		
	**(*n* = 828)**		**(*n* = 699)**		**(*n* = 129)**		
**Maternal Characteristics**	*n*	%	*n*	%	*n*	%	*p* [1]
Maternal age at birth (years)							0.948
<25	73	8.8	62	8.9	11	8.6	
25–29	260	31.5	221	31.7	39	30.5	
≥30	492	59.6	414	59.4	78	60.9	
Maternal education completed							0.057
School/vocational	355	43.1	310	44.5	45	35.4	
Some university and above	468	56.9	386	55.5	82	64.6	
IRSAD score [2]							0.983
Deciles 1–2	120	14.6	102	14.7	18	14.0	
Deciles 3–4	173	21.0	145	20.9	28	21.7	
Deciles 5–6	172	20.9	146	21.1	26	20.2	
Deciles 7–8	161	19.6	137	19.8	24	18.6	
Deciles 9–10	196	23.8	163	23.5	33	25.6	
Mother's country of birth							0.404
Australia and New Zealand	611	74.2	511	73.4	100	78.1	
Asia, other	46	5.6	40	5.7	6	4.7	
India	50	6.1	43	6.2	7	5.5	
China	37	4.5	35	5.0	2	1.6	
UK	31	3.8	24	3.4	7	5.5	
Other	49	5.9	43	6.2	6	4.7	
Maternal BMI (kg/m^2)							0.549
<25	477	60.9	407	61.2	70	59.3	
25–29.99	167	21.3	144	21.7	23	19.5	
>30	139	17.8	114	17.1	25	21.2	
Parity	826						0.483
Primiparous	414	50.1	353	50.6	61	47.3	
Multiparous	412	49.9	344	49.4	68	52.7	
**Child Characteristics**							
Child age in months (mean SD)	12.80	0.82	12.85	± 0.84	12.80	± 0.70	<0.001 [3]
Infant sex							0.283
Boy	453		388		65		
Girl	375		311		64		

[1] Chi Square p value, [2] Index of Relative Socio-Economic Advantage and Disadvantage (IRSAD) where decile 1 = most disadvantaged and decile 10 = most advantaged, [3] Independent *t*-test. SD: standard deviation; BMI: body mass index.
